# Cationic antimicrobial peptides: potential templates for anticancer agents

**DOI:** 10.3389/fmed.2025.1548603

**Published:** 2025-04-24

**Authors:** Yahson Fernando Varela-Quitián, Fabio Enrique Mendez-Rivera, David Andres Bernal-Estévez

**Affiliations:** Immunology and Clinical Oncology Research Group (GIIOC), Fundación Salud de Los Andes, Bogotá, Colombia

**Keywords:** cationic antimicrobial peptides (CAMPs), anticancer peptides (ACPs), peptide-based cancer therapeutics, synthetic peptide design, proteolytic stability enhancement, computational peptide engineering, clinical translation

## Abstract

Cancer is a major global health concern and one of the leading causes of death worldwide. According to the World Health Organization (WHO), there is an urgent need for novel therapeutic agents to treat this disease. Some antimicrobial peptides (AMPs) have demonstrated activity against both microbial pathogens and cancer cells. Among these, cationic AMPs (CAMPs) have garnered significant attention because of their ability to selectively interact with the negatively charged surfaces of cancer cell membranes. CAMPs present several advantages such as high specificity for targeting cancer cells, minimal toxicity to normal cells, reduced probability of inducing resistance, stability under physiological conditions, ease of chemical modification, and low production costs. This review focuses on CAMPs with anticancer properties such as KLA, bovine lactoferricin derivatives, and LTX-315, and briefly explores common bioinformatics tools for Anticancer Peptides (ACPs) selection pipeline from AMPs.

## 1 Introduction

Cancer is one of the leading causes of mortality worldwide, according to the World Health Organization (WHO). In 2022, global statistics reported nearly 20 million new cancer cases and 9.7 million cancer-related deaths. Over the past 40 years, the number of deaths due to cancer has doubled in women and tripled in men (1, 2). This public health problem is closely related to the increase and aging of the global population (3).

Cancer treatment approaches that attempt to kill cancer cells directly, such as surgery, radiotherapy, chemotherapy, or combinations thereof, are frequently used (4). Surgery is only effective for localized tumors; however, incomplete removal of cancerous tissues increases the risk of recurrence (5). Radiotherapy specifically targets tumors, but has adverse effects on the surrounding healthy tissues, and its efficacy varies with the type and location of the tumor (6). Chemotherapy is one of the most widely used mode of treatment, but it is toxic to both cancer and healthy cells, and cancer cells may become resistant over time, making the treatment progressively less effective (7). In recent years, immunotherapy approaches that try to help the immune system fight cancer have been developed. However, tumor complexity, high resistance to solid tumors, and the ability of certain cancer cells to evade anticancer drugs result in a low percentage of treatment-surviving patients (8). These challenges highlight the urgent need for novel therapeutic agents (9–11).

Anticancer peptides (ACPs) are currently being explored as new alternatives to conventional cancer therapies, with the potential to selectively kill cancer cells. ACPs do not depend on unique receptors or a specific signal transduction pathway for their action, making it more difficult for tumors to develop resistance (12).

Many ACPs are derived from antimicrobial peptides (AMPs), particularly cationic AMPs (CAMPs), which bind selectively to the negative charged membrane of cancer cells. The membrane integrity and the mitochondrial function are compromised by CAMPs resulting in apoptosis or necrosis, thus CAMPs are promising candidates for anticancer therapy (13–17).

This review explores the potential applications of CAMPs derived from natural or artificial sources and examines how their unique characteristics can serve as inspiration for the development of novel anticancer agents. Notable examples of the use of CAMPs as templates for producing anticancer agents include derivatives that have improved the design of KLA, bovine lactoferricins, and LTX-315 peptides. Additionally, it briefly presents computational tools employed in the pipeline for selecting ACPs from AMP.

## 2 AMPs classified by charge

**FIGURE 1:**
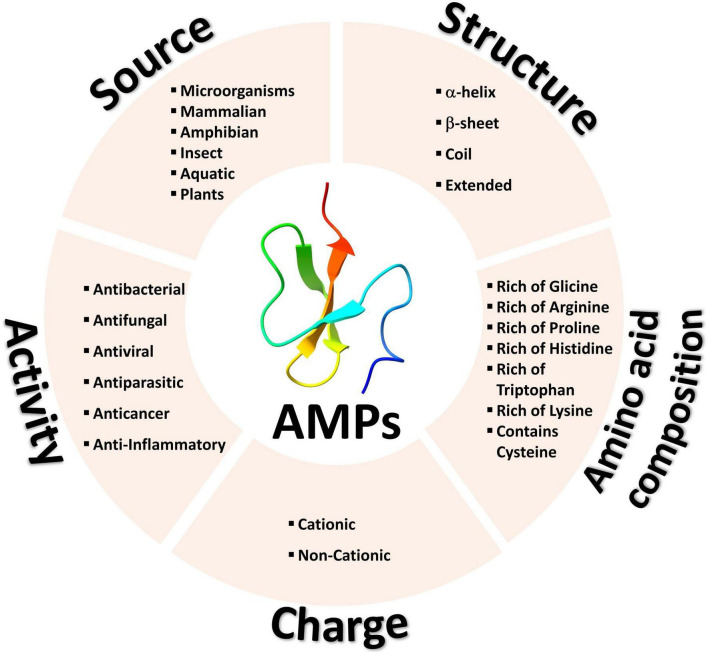
Common methods for classifying antimicrobial peptides.

Antimicrobial peptides can be classified into two main groups based on their charge, cationic and non-cationic peptides (Figure 1). Each group can include peptides from diverse sources with varying activities, structures, and amino acid compositions (18–20).

CAMPs constitute nearly 90% of all AMPs and carry a positive net charge, enabling an initial interaction with the negatively charged microbial cell membrane (21). Notably, some CAMPs also exhibit anticancer activity, selectively targeting tumor cells while sparing normal cells. Similar to their electrostatic interaction with microbial cell membranes, CAMPs engage with tumor cells owing to their more negative charge compared to normal cells (22, 23). Due to this fact, CAMPs are considered as potential tool for cancer therapy and have been used in pharmaceutical sciences, drug design and discovery (24, 25), and cancer clinical trials (26–28).

## 3 Mode of action of CAMPs with anticancer activity

The membrane composition of tumor cells is considerably different and more anionic than the membrane of normal cells, which enables CAMPs to interact with the tumor membrane, similar to the microbial membrane (21). Two mechanisms of action have been identified for CAMPs against cancer cells: membrane- and non-membrane-targeting mechanisms (29).

**FIGURE 2:**
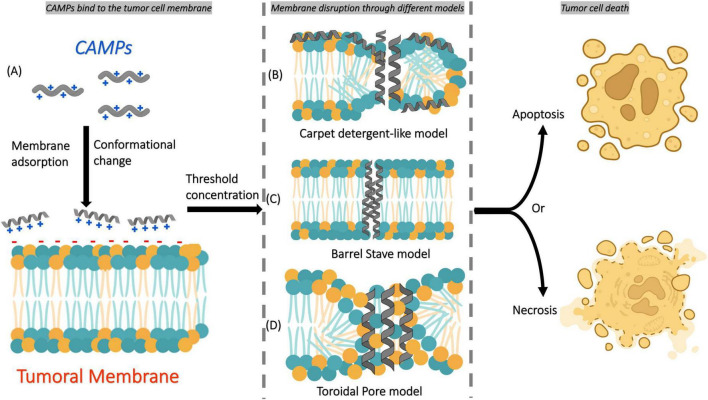
Simple scheme of the main mechanism of action of CAMPs on tumor cell membranes. **(A)** CAMPs first bind to the tumor cell membrane through electrostatic interactions and become adsorbed onto its surface, inducing conformational changes in their structure. Once a threshold concentration is reached, membrane disruption occurs through different mechanisms: **(B)** carpet detergent-like model, **(C)** barrel-stave model, or **(D)** toroidal pore model. These disruptions ultimately lead to tumor cell death by apoptosis or necrosis, depending on the mechanism involved.

The first mechanism involves positively charged amphipathic CAMPs that interact with negatively charged cell membranes. Then, they are adsorbed into the membrane, which results in a conformational change and membrane disruption according to different models, including carpet detergent-like, barrel-stave (formation of transmembrane pores), and toroidal (lipid rearrangement for pore formation) (Figure 2) (30, 31).

The second mechanism involves CAMPs that penetrate the cells directly or by endocytosis. Inside the cytoplasm, they target important cellular structures and processes by inhibiting protein biosynthesis, nucleic acid biosynthesis, protease activity, and cell division (15).

These interactions, which are described by the two mechanisms of action, ultimately leading to membrane lysis or pore formation, which triggers apoptosis or necrosis in tumor cells with minimal impact on normal cells (25).

## 4 Physicochemical factors influencing the anticancer activity of CAMPs

### 4.1 Attributes of CAMPs

Several physicochemical factors, such as amino acid composition, net charge, hydrophobicity, amphipathicity, structural folding, peptide concentration, oligomerization, and membrane composition, affect the anticancer activity of CAMPs (32, 33). Understanding the relationship between the peptide sequence and function is crucial for the rational design of novel CAMP-based therapeutics with improved anticancer efficacy.

#### 4.1.1 Amino acid composition

A systematic analysis of amino acid-rich AMPs from the Antimicrobial Peptide Database (APD) as well as their distribution across different life kingdoms and animal classes, reveals a low abundance of Asp, Glu and Met; a moderate abundance of Phe, Ser, Thr, Trp and Tyr; and a high abundance of Ala, Cys, Gly, His, Ile, Lys, Leu, Pro, Arg and Val (32, 33).

**FIGURE 3:**
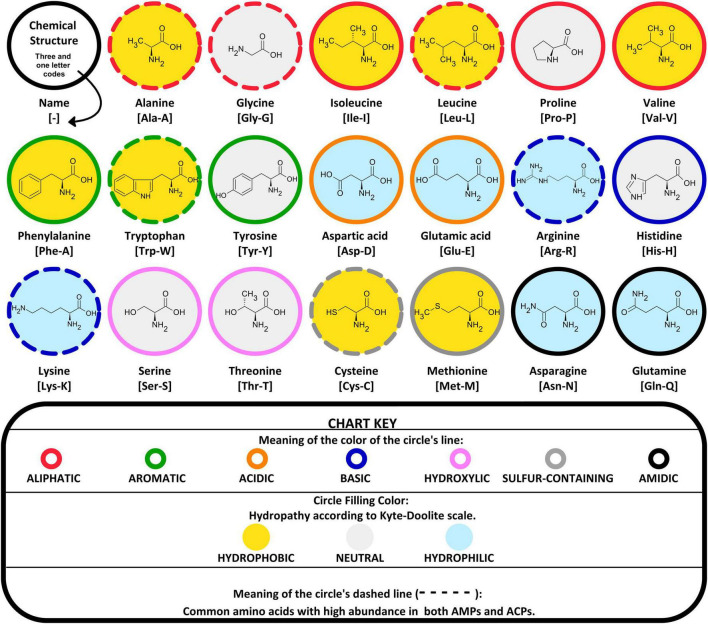
Grouping of 20 common amino acids. Based on their chemical characteristics and hydropathy, they are derived from the physicochemical properties of the amino acid side chains. Additionally, the circle dashed line corresponds to amino acids with high preference for both AMPs and ACPs.

In general, AMPs are characterized by a high prevalence of amino acid residues classified as hydrophobic, cationic (basic), and aromatic (32–38). ACPs derived from AMPs were expected to show a similar trend in amino acid abundance (Figure 3). In silico models and exploratory data analysis confirmed a preference for certain amino acids (Ala, Cys, Gly, Lys, and Leu) in both AMPs and ACPs global datasets (39, 40). Additionally, Arg and Trp amino acids were also found in many AMPs (20, 41–43) and ACPs (44, 45).

#### 4.1.2 Net positive charge

AMPs are generally cationics (CAMPs), with their charge varying from +2 to +11, owing to the overrepresentation of Arg and/or Lys residues. It is widely accepted that cationicity conferred by Arg and/or Lys is primarily responsible for the initial interaction of CAMPs with the negatively charged membrane surface of cancer cells. Studies have demonstrated a direct correlation between the peptide charge and cytotoxicity against tumor cells (46–48).

In addition, these basic amino acids (charged Arg or Lys) can deform the lipid bilayer by pulling water molecules and lipid head groups into the hydrocarbon core of the lipid membrane, as demonstrated by atomistic molecular dynamics. However, Arg causes greater membrane perturbations by attracting more lipid phosphate groups because Arg forms more hydrogen bonds with lipid phosphates than Lys (49).

In general, peptides enriched in Lys within their hydrophilic regions exhibits more selective anticancer activity, whereas those containing Arg tend to display higher toxicity in normal cells (50), as observed with AMP/ACP tritrpticin (RRFPWWWPFLRR) when arginine was substituted with lysine, which improved the selectivity of peptides toward Jurkat cancer cells compared with normal peripheral blood mononuclear cells (PBMCs) (51).

#### 4.1.3 Amphipathicity

Amphipathicity refers to the spatial distribution of hydrophilic and hydrophobic peptide residues, which allows AMPs to align themselves at the membrane interface, with hydrophobic residues facing the lipid core and hydrophilic residues interacting with the aqueous environment (52, 53).

The initial interaction between CAMPs and microbial or tumor membranes is driven by electrostatic forces through the interaction of the positively charged hydrophilic region of CAMP and negatively charged components of these membranes; subsequently, the hydrophobic region of CAMPs becomes embedded in the membrane through van der Waals interactions. This leads to compromised membrane functionality and increased permeability (54–57).

#### 4.1.4 Hydrophobicity

Hydrophobicity, an important physicochemical characteristic of AMPs, is the percentage of hydrophobic amino acids in the peptide. Typically, AMPs have hydrophobicity values close to 50%, and are expected to contain both hydrophilic and hydrophobic amino acid residues (58). AMPs can interact with membranes, and certain ratios of hydrophilic charged residues affect peptide-membrane interactions (59) as they control the partitioning of the peptide into the membrane hydrophobic core (60).

Peptides can adopt different structures based on hydrophobicity and environmental factors, such as folding into an α-helical conformation in the presence of certain micelles (61). Manipulation of peptide hydrophobicity can enhance anticancer activity by altering their amphipathicity and secondary structure conformation. Studies indicate that increasing the hydrophobicity of peptides may increase their self-association and cytotoxicity against cancer cells (62), but sometimes also increase hemolytic activity (61, 63).

For example, the N-terminus of AMP CM4 (RWKIFKKIEKVGQNIRDGIVKAGPAVAVVGQAATI) was modified by conjugating fatty acids of varying lengths (4–16 carbons) to enhance hydrophobicity, helical content, and anticancer activity. The best anticancer effects were observed in breast cancer cells when AMPs were conjugated with fatty acids of 12–16 carbons (64). Hence, hydrophobicity is a critical parameter that must be optimized for the development of CAMP/ACP-based drugs.

#### 4.1.5 Structural folding

The conformation of a peptide structure can be affected by the environment in which it is immersed. In aqueous solutions, linear AMPs are mostly unstructured; however, upon interaction with the hydrophobic environment of the lipid bilayers, they undergo significant conformational changes. By adopting specific secondary structures, such as α-helices, β-sheets, or extended polyproline-like helices, which increase amphipathicity, this structural flexibility allows AMPs to adapt to various targets (53).

ACPs may adopt α-helical, β-sheet, or linear conformations, with α-helical ACPs representing one of the largest groups recognized (65, 66). α-helical AMP/ACP showed higher activity than linear peptides, which has been attributed to the fact that the α-helical conformation can project a clear hydrophobic surface and another hydrophilic surface that allows effective interaction with microbial and tumor membranes.

#### 4.1.6 Peptide concentration

Antimicrobial peptides with anticancer activity display concentration-dependent cytotoxicity toward cancer cells and solid tumors in various studies conducted (57). Another important feature is the behavior of AMP monomers in solution and their proximity to the cancer cell membrane, which facilitates membrane aggregation and subsequent pore formation, leading to cell death (65). For example, to induce lytic activity, the cancer cell membrane needs to be exposed to a minimum threshold concentration of monomers, termed the critical concentration, and this concentration-dependent process is critical for the therapeutic efficacy of AMPs (23).

#### 4.1.7 Oligomerization

The capacity for peptide self-association, also known as oligomerization, is an important characteristic of AMPs. This oligomerization process is dependent on the amino acid composition, conformation, and ability of the peptide to align hydrophobic and hydrophilic regions toward adjacent peptides and the membrane (67).

### 4.2 Membrane composition of cancer cells

Cancer cells differ from normal cells, which is important for understanding the anticancer activity of CAMPs and their interaction with tumor cells. The initial recognition between CAMPs and cancer cells is mainly due to electrostatic interactions. Cancer cells are more negatively charged than normal cells due to increased expression of anionic molecules. In addition, cancer cells have different cholesterol content, microvilli structure, and extracellular pH, which also affect CAMP interactions and selectivity (25, 68).

#### 4.2.1 Negative charge

Cancer cell surfaces are more negatively charged due to the increased expression of anionic molecules such as phosphatidylserine (PS), O-glycosylated mucins, and heparan sulfates (HS), and their exposure to cancer cells, which improves their interaction with CAMPs (68).

In normal cells, PS is found on the surface of the interior membranes, whereas in cancer cells, it is externalized, producing an immune suppressor environment that can promote tumor growth (69–71), the externalization of PS is a universal feature of cancer cells, as observed in tumor endothelial cells (ECs) (72). In addition, PS overexpression has been observed in some cancer cells, including glioblastoma (Gli), astrocytoma (U373), and breast cancer (MDA-MB-231-D3H2LN) (70).

In cancer cells, anionic molecules derived from O-glycosylated mucins are primarily obtained by sialylation and sulfation, which involve the addition of sialic acid and/or sulfate groups, respectively. O-glycosylated mucins are crucial for cancer progression, metastasis, and immune evasion (73).

HS is a type of glycosaminoglycan; it is an unbranched chain of disaccharide repeats that is heavily sulfated at various positions on their sugar residues. HS can modulate the effects of various growth factors and is involved in angiogenesis and metastasis of cancer cells, and its overexpression has been reported in several tumors (74–76). HS serves as an initial anchor for cell-penetrating peptides (CPPs) via electrostatic interactions between the sulfates or carboxylic groups of HS and the basic amino acids (Arg and/or Lys) of CPPs (77).

#### 4.2.2 Cholesterol content

Cholesterol is a key part in the formation of cell membranes and relevant component in maintaining the integrity and organization of the of phospholipid bilayers (78). Cholesterol content affects the regulation of membrane fluidity and modulates the chemotherapy resistance and metastatic properties of cancer cells. When cancer cells prepare for metastasis, they tend to decrease their membrane cholesterol levels to maximize membrane fluidity and plasticity, allowing neoplastic cells to modulate their shape easily (79).

Notably, leukemia and lung cancer cells are more fluid than healthy cells, owing to their lower cholesterol levels. In contrast, the opposite trend was observed in breast and prostate cancer cells (65). Malignant cells with reduced cholesterol content are more susceptible to lysis, which can facilitate AMP-induced apoptosis (80, 81).

#### 4.2.3 Microvilli

A notable difference between cancer and normal cells is the significantly higher number of microvilli in cancer cell membranes, which increases their surface area (68). This structural change is believed to affect the action of AMPs, as cancer cells increase their membrane fluidity and microvilli density. The increased number of microvilli augments surface contact and peptide attraction, making cancer cells more sensitive to AMP interactions. Consequently, this positive feedback mechanism enhances the efficacy of AMPs against cancer cells while reducing the risk of resistance development compared with traditional chemotherapy approaches (65, 80).

#### 4.2.4 Extracellular pH acidification

Cancer tissues have a hypoxic environment caused by limited blood supply; under these conditions, cancer cells elevate their production of carbonic anhydrase IX, an enzyme that catalyzes the reversible conversion of carbon dioxide to bicarbonate and a proton, referring to its contribution to an acidic microenvironment, so the extracellular pH (pH_*e*_) of cancer cells is lower (pH 6.2–6.9) than that of normal cells (pH 7.3–7.4) (68).

This phenomenon has been well documented, as shown by the Guanyu Hao group, who used data collected from several different sources to compare the average extracellular pH_*e*_ values of different normal tissues with those of cancerous tissues (malignant melanoma, vulvar tumor, urine tumor, lung tumor, breast tumor, glioblastoma, astrocytoma, and sarcomas). Their findings make evident the tendency of the higher acidity of the extracellular tumor environment over normal cells, showing 0.3–0.7 pH units lower of pH_*e*_ of cancer cells than normal cells (82). This lower extracellular pH_*e*_ has been exploited to induce selective toxicity in cancer cells using peptide conjugates (83–85).

## 5 Cationic antimicrobial peptides with antitumor properties

Chemotherapy remains the predominant treatment for controlling tumor cells, many anticancer drugs lack specificity and affect cancer and normal cells, causing toxic side effects. Furthermore, tumor cells are prone to developing resistance, which further diminishes the effectiveness of treatment (86).

Given these challenges, the development of new therapeutic approaches which specifically attack cancer cells without damaging normal tissues represents an essential requirement (24). In this context, the exploration of novel CAMPs as prospective anticancer agents has emerged as a significant field.

### 5.1 KLA

KLA peptide **(KLAKLAK)**_2_ is a *de novo*-designed CAMP developed by Javadpour et al. in 1996, and it was designed to adopt an amphipathic helical conformation. Initially, its antibacterial activity was evaluated against Gram-negative Bacteria *Escherichia coli* and *Pseudomonas aeruginosa*, and Gram-positive bacteria *Staphylococcus aureus*, with minimal inhibitory concentrations (MICs) of 6, 3, and 6 μM, respectively. In contrast, KLA exhibited low cytotoxicity against 3T3 mouse fibroblasts (>517 μM) and human erythrocytes, suggesting a favorable safety profile (>750 μM) (87).

Subsequently, KLA was evaluated *in vitro* against various human cancer cell lines (breast, prostate, and bladder cancer cells) and *in vivo* in a breast cancer xenograft model in mice. The results demonstrated its potent anticancer effects, including of cell death in most of the cancer cell lines tested. Among them, breast cancer cell line MCF-7 exhibited the highest sensitivity to KLA (Table 1), while the peptide had minimal effects on healthy control cells (peripheral blood lymphocytes and embryonic kidney epithelial cells). Furthermore, KLA significantly inhibited tumor growth and prolonged the overall survival of tumor-bearing mice compared with that of the control group (88).

**TABLE 1:** Cytotoxic effects of some representative CAMPs with anticancer activity evaluated *in vitro* in different cancer cell lines.

Name	Sequence	Source	Charge	Cell lines-type	IC_50_ (μM)	References
**KLA (KLAKLAK)**_2_	KLAKLAKKLAKLAK	De novo	+6	**Breast cancer**		(88)
				MCF-7	88.1	
				MDA-MB435S	140.0	
				MDA-MB435	191.0	
				SKBR3	>320	
				T47D	247.0	
				**Prostate**		
				DU145	183.3	
				**Urinary bladder carcinoma**		
				T24	161.6	
				Neuroblastoma*		
				Tet21N	167.1	
				Wac2	181.3	
				PBL*	>320	
				293*	>320	
**Lactoferricin (LfcinB)**	FKCRRWQWRMKK LGAPSITCVRRAF	Milk	+8	**Gastric cancer**		(101)
				AGS	64	
**LfcinB11**	RRWQWRMKKLG	Milk	+5	**Gastric cancer**		(101)
				AGS	>500	
**LfcinB6**	RRWQWR-OH	Milk	+3	**Gastric cancer**		(101)
				AGS	>500	
**LTX-315 (Oncopore)**	KKWWKKW-Dip-K	De novo	+6	**Lung**		(122)
				A549	2.8	
				Calu-6	2.1	
				MRC-5*	9.2	
				NCI-H460	3.4	
				**Colon**		
				COLON 205	2.6	
				HCT-116	3.4	
				HCT-15	3.8	
				HT-29	3.3	
				**Liver**		
				Hep G2	6.7	
				SK-HEP-1	5.1	
				**Breast cancer**		
				MCF-7	2.2	
				MCF-7/mdr	2.5	
				MDA-MB4231	3.6	
				MDA-MB435S	3.1	
				T47D	1.2	
				**Prostate**		
				DU 145	3.9	
				PC-3	4.4	
				**Skin**		
				A-431	3.2	
				Malme-3M	3.3	
				SK-MEL-2	5.2	

*PBL, 293, and MRC-5 are not cancer cell lines and were used as non-cancer controls.

Jeffrey et al. designed DP1 (RRQRRTSKLMKRGG**KLAKLAKKLAKLAK**), a conjugated tumor targeting peptide. DP1 consists of an N-terminal fragment derived from Protein Transduction Domain-5 (PTD-5), which facilitates the delivering protein complexes into solid tumors, and C-terminal KLA sequence. When DP1 was directly added at low concentrations to the culture medium of the mouse fibrosarcoma cell line MCA205, *the results showed a significant reduction of cell viability (LC_50_ < 50 μM) and triggered significant apoptosis (programmed cell death) *in vivo*; in contrast, no significantly effect of KLA or PTD-5 alone were observed on MCA205 viability (89).

Similarly, a targeted cancer-killing peptide named TP-tox (LTVSPWYGG**KLAKLAKKLAKLAK**) was developed to mimic antibody-drug conjugates (ADCs) without the size restrictions of conventional ADCs. TP-Tox exhibited selective toxicity in breast, prostate, and neuroblastoma cancer cell lines. It was more effective at killing cancer cells than the individual targeting or killing peptides components. Additionally, weekly injections of TP-ox significantly slowed tumor growth and improved survival in mice with breast cancer tumors (MDA-MB-435S) (90).

A set of pH-dependent targeting KLA-conjugated peptides was developed to prevent off-target effects. It was conducted using a pH-low insertion peptide (pHLIP) and three KLA analogs. pHLIP peptides undergo conformational changes in acidic microenvironments and promote peptide translocation across the cytoplasmic membrane, such as in tumor microenvironments (29). Therefore, these conjugated peptides can translocate KLA to the cytoplasm of breast cancer cells. Each KLA was synthesized individually with a cysteine residue at the N-terminus, while pHLIP, containing a cysteine at the C-terminus (GGEQNPIYWARYADWLFTTPLLLLDLALLVDADEGTCG), was conjugated to each KLA analog by disulfide bonds and purified. The resulting chimeric peptides [pHLIP-**(KLAKLAK)**_2_, pHLIP-**KLAKLAK**, or pHLIP-**KLAK**] were evaluated in MDA-MB-231 cancer cells and showed concentration- and pH-dependent cell growth inhibition, with little or no significant decrease in cell viability at pH 7.4, and 90% inhibition at pH 5.0. pHLIP-**KLAKLAK** was identified as a lead conjugate with a IC_50_ of 0.5 μM to MDA-MB-231 cancer cells (84).

Several other conjugated peptides incorporating the KLA fragment have been tested as potential cancer therapeutics, showing promising results (11, 91–93). Additionally, KLA-conjugated peptides with replacement of all L-to D-amino acids in the KLA fragment (D-KLA) were evaluated. These D-KLA peptides retain membrane-disrupting properties similar to those of their L-counterparts but exhibit enhanced stability in biological systems (94–96).

### 5.2 Bovine lactoferricin

Bovine lactoferricin (LfcinB) is 25-amino acids peptide (^17^**FKCRRWQWRMKKLGAPSITCVRRAF**^41^) generated from enzymatic digestion of the N-terminal region of bovine lactoferrin. Its sequence contains eight hydrophilic residues (five arginine and three lysine), conferring a net charge of +8, along with four aromatic and hydrophobic residues (two phenylalanine and two tryptophan), and two cysteines forming disulfide bond. In aqueous solution, LfcinB adopts a β-sheet secondary structure, where hydrophobic residues are positioned on one side and hydrophilic residues on the opposite side (97).

Due to its cationic and amphipathic nature, LfcinB interacts with negatively charged molecules on cell surfaces and exhibits broad-spectrum antimicrobial activity against Gram-positive and Gram-negative bacteria, fungi, and viruses, primarily by interrupting the microbial cell membranes. LfcinB also exhibits anticancer properties by selectively targeting negatively charged cancer cell surfaces, leading to apoptosis or necrosis (57, 98).

LfcinB has demonstrated cytotoxic activity *in vitro* in various human and murine cancer cell lines, including colon carcinoma, lung cancer, liver cancer, melanoma, fibrosarcoma, leukemia, and breast cancer. Notably, LfcinB treatment does not significantly affect the viability of normal human lymphocytes, erythrocytes, endothelial cells, or fibroblasts (99). Additionally, many synthetic peptides derived from LfcinB exhibit anticancer properties, highlighting their potential as novel therapeutic agents for cancer treatment (100).

A study by Pan et al. evaluated bovine lactoferricin peptide fragments (Table 1) in AGS gastric cancer cells. The assays revealed that full-length LfcinB peptide selectively inhibited AGS cell proliferation in a dose-dependent manner, with a half-maximal inhibitory concentration (IC_50_) of 64 μM. Furthermore, treatment with LfcinB results in an increased sub-G1 population within the cell cycle, indicating the induction of apoptosis (101).

#### 5.2.1 Synthetic derivates inspired by LfcinB

The hexapeptide LfcinB6 (**RRWQWR-NH**_2_), which contains a C-terminal carboxamide functional group, retains the antimicrobial activity of LfcinB (102–105). However, unlike native LfcinB, LfcinB6 lacks inherent cytotoxic activity against T-leukemia or breast cancer cells. This difference is attributed to weak binding to isolated mitochondria, which prevents membrane permeabilization or causes cytochrome C release. Interestingly, when LfcinB6 was delivered using fusogenic liposome formulations, it was efficiently transported into the cytosol of cancer cells, where it exhibited strong cytotoxic activity. The mechanism of cytotoxicity was found to be caspase-and cathepsin B-dependent but not reliant on reactive oxygen species (ROS), suggesting a mode of action distinct from that of native LfcinB (106).

To further enhance the anticancer activity of LfcinB6, it was conjugated to CPP peptide containing seven arginine residues, resulting in MPLfcinB6 (RRRRRRRGG**RRWQWR**). This modified peptide demonstrated selective cytotoxicity against T-leukemia and B-lymphoma cells, while sparing normal T cells. MPLfcinB6 rapidly induced extensive cancer cell membranes damage, triggered ROS production, and disrupted mitochondrial integrity. Its high selectivity was attributed to strong electrostatic interaction between its highly cationic seven-arginine motif and the negatively charged membranes of cancer cells (107).

Inspired by the core sequence of LfcinB6, which contains cationic terminal residues, a lipophilic central core, and a well-defined amphipathic structure upon interaction with negatively charged micelles, Torfoss et al. synthesized eight heptapeptides (**H-KKWβ** ^2,2^**WKK-NH**_2_), each with different central lipophilic β^2,2^-amino acid building blocks. Among these, one peptide demonstrated notable anticancer activity, with IC_50_ values 23 μM against Ramos cancer cells and 22 μM against A20 carcer cells. Additionally, this peptide exhibited a high selectivity index, low toxicity, and improved protein stability (108). Notably, the promising peptide contains two p-trifluoromethyl benzylic substituents within its β^2,2^ amino acid side chain. These modifications are known to enhance pharmacokinetic properties, alter the electronic distribution of aromatic side chains, and increase lipophilicity, thereby optimizing peptide interactions with the cell membrane components (109–111).

### 5.3 LTX-315

Rekdal et al. designed, synthesized, and screened a series of peptide inspired by LfcinB derivatives, which demonstrated preferential cytotoxicity against cancer cells over normal cells (112–115). The culmination of their efforts led to the development of a novel and optimized CAMP/ACP named LTX-315 (**KKWWKKW-Dip-K**) (116).

LTX-315 was obtained by screening a set of lytic nonapeptides using the sequence template **KKWWKKWWK**, in which tryptophan (Trp) residues were systematically substituted with non-coding amino acids: Ath: 9-anthracenylalanine, Bip: Biphenylalanine, Dip: 3,3-diphenylalanine, 1-Nal: 1-naphthylalanine, and 2-Nal: 2-naphthylalanine. The KKWWKKWWK peptide has a net charge of +6 at physiological pH and is modeled as an α-helical amphipathic structure, with aromatic residues on one side of the helix and cationic residues on the other. Notably, the incorporation of non-coding amino acids has allowed the production of CAMPs (117) and CAPs, enhancing their structural diversity and functional properties (118–121).

Initially, 18 peptides, template peptide and 17 modified peptides, were tested against human A20 lymphoma and murine AT84 squamous cell carcinoma cell lines. A lead series of five peptides, including LTX-315, were identified. These lead candidates were subsequently evaluated against a broad range of cancer cell lines, including drug-resistant strains, were LTX-315 emerged as a highly effective candidate with low activity toward healthy cells (Table 1). Consequently, LTX-315 peptide was selected as the lead candidate for preclinical and clinical studies (122).

LTX-315 is not harmful to human red blood cells (hRBCs), with EC_50_ > 695 μM. In contrast, it has been observed to rapidly induce cancer cell death *in vitro*, triggering the release of several danger signals patterns (DAMPs) associated with immunogenic cell death (ICD) and enhanced adaptive immunity. Additionally, LTX-315 induces inflammation and activation of immune cells, such as cytotoxic CD8+ T cells, resulting in tumor shrinkage and systemic immune reactions, as shown in preclinical studies (119, 122, 123).

Two notables preclinical studies explored the combination of LTX-315 with doxorubicin or anti-CTLA-4 antibody. First, administration of LTX-315 with CAELYX^®^ (bran name of the chemotherapy drug doxorubicin) in a murine model triple-negative breast cancer (TNBC) yielded promising results. 4T1 breast cancer cells were implanted, which subsequently mice received LTX-315, CAELYX, or a combination of both. The combination therapy significantly reduced the tumor size, leading to complete regression in 50% of the cases. It also causes extensive tumor necrosis and increases the infiltration of CD8+ T cells into tumors (124). The second study involved the combination of LTX-315 and anti-CTLA-4 antibody, which block the interaction of CTLA-4 with its ligands B7.1 and B7.2 to enhance immune responses, including antitumor immunity. In murine models of sarcoma and melanoma, LTX-315 and anti-CTLA-4 were injected directly into tumors and responses in both treated and untreated tumors, as well as changes in immune cells, were monitored. The combination therapy proved more effective in reducing and eliminating tumors than individual treatments; where LTX-315 decreases the population of suppressor cells and increases active cancer-fighting cells, altering the tumor environment, whereas anti-CTLA-4 enhances the immune response (125). These two promising preclinical studies were conducted in animal models, and further research is required to evaluate the safety and efficacy of these combination therapies in clinical trials.

In a phase I clinical trial (NTCNCT01986426), LTX-315 was evaluated in a dose-escalation study involving intratumoral administration, to assess its safety, tolerability, and efficacy. A total of 39 patients with various advanced solid tumors, including melanoma, breast, head and neck, sarcoma, gastrointestinal, desmoid, pancreas, primary vaginal cancer, and carcinoma of unknown primary, were enrolled. These results indicate that LTX-315 has an acceptable safety profile, is clinically active, induces alterations in the tumor microenvironment, and promotes immune-mediated anticancer activity (126).

Currently, a phase II clinical trial (NCT04796194) is being conducted to investigate the use of LTX-315 in combination with pembrolizumab, focusing on patients with advanced melanoma who have access to percutaneous injections (127). Pembrolizumab is a human antibody approved by the FDA for use in cancer immunotherapy to bind to and block the PD-1 receptor on lymphocytes. It is an immune checkpoint inhibitor (ICI) that avoids the mechanisms used by many cancer cells expressing PD-L1 on their surfaces that could interact with PD-1 in T cells and send a signal to deactivate T cells, effectively preventing them from attacking the tumor (128). To date, LTX-315 has undergone six clinical trials (NCT01986426, NCT01223209, NCT03725605, NCT05188729, NCT01058616, NCT04796194), demonstrating its great potential and versatility as a short cationic peptide with ACP properties.

#### 5.3.1 NTP-217: a synthetic derivate inspired by LTX-315

A derivative of LTX-315 conjugated to rhodamine B (NTP-217) exhibited remarkable enhancement in anticancer activity, achieving a 2.4 to 37.5-fold increase in potency across a diverse panel of adherent cancer cell lines (129). Notably, the efficacy of the hybrid peptide NTP-217 (H-rhodamine **B-GABA-KKWWKKWDipK-NH**_2_, where GABA refers to gamma-aminobutyric acid) was rigorously evaluated against liver cancer cells in both *in vitro* and *in vivo* models.

Comprehensive assessments, including cell proliferation, cell migration assays, as well as *in vivo* tumor growth experiments, confirmed NTP-217’s potent anticancer effects. The study concluded that NTP-217 significantly outperformed the parent peptide LTX-315, which proved to be more potent in inhibiting proliferation and migration of liver cancer cells (130).

## 6 Chemical modifications to enhance peptide resistance to proteolytic degradation

Peptides, particularly AMPs, offer remarkable versatility in cancer treatment and possess substantial potential for overcoming the shortcomings of alternative therapeutic methods (11, 131). However, a key challenge in developing peptide-based medications is that AMPs, which are sometimes effective *in vitro*, often lose their activity *in vivo* because of proteolytic degradation in serum (132).

To prevent or mitigate proteolytic degradation, several chemical modification strategies have been employed in the development of synthetic antimicrobial peptides, such as terminal protection, backbone modification, glycosylation, PEGylation, and cyclization (17, 63, 133, 134).

### 6.1 Terminal protection

Terminal protection strategies help to protect peptides against proteolytic degradation by peptidases, which can cleave both the N-terminal and C-terminal regions of the peptide. This chemical modification can also enhance the half-life and therapeutic efficacy. N-terminal acetylation or C-terminal amidation can fulfill this purpose (135) and similar objectives can be achieved by modifying the termini with unnatural amino acid analogs (136).

### 6.2 Backbone modification

Backbone modifications include substitution reactions, such as replacing the carbonyl oxygen with sulfur or substitution hydrogen atoms in nitrogen or α-carbon. D-amino acids can be considered a form of backbone modification, and their incorporation into the peptide backbone can increase the resistance to proteolytic degradation. These modifications can also produce profound changes in molecular chirality and conformation, thereby improving peptides stability and bioavailability (17, 137, 138).

### 6.3 Glycosylation

Glycosylation is the peptide-carbohydrate bond formation which is utilized to improve the cancer treatment outcomes. It improves the tissue targeting, enhances the delivery to the tumor and extends the serum half-life through the inhibition of enzyme degradation. Glycosylated peptides generally show greater permeability across the membrane, which leads to increased cellular uptake and reduced off-target toxicity. Furthermore, glycosylation can affect the immunogenicity of the peptide, which enhances the biocompatibility and the *in vivo* circulation time. The performance of glycosylated peptide conjugates is a function of the arrangement, type, and number of sugar units which affect the stability, cellular interaction, and therapeutic potential of these compounds (134).

### 6.4 PEGylation

PEGylation is the attachment of one or more chains of polyethylene glycol (PEG) to a peptide. The application of this modification leads to an increase in peptide solubility and an inhibition of renal clearance together with protection against enzymatic degradation (139), resulting in an extension of the half-life and increased systemic circulation (140). The use of PEGylated peptides has been shown to produce decreased immunogenicity along with enhanced tumor penetration properties, resulting in sustained drug release and improved bioavailability (141).

### 6.5 Cyclization

Cyclization enhances peptide stability and bioactivity by reducing conformational flexibility and providing better protection against degradation. The formation of a covalent bond, either at the termini or side chains, increases structural rigidity that results in a longer half-life. The anticancer potency of AMPs has been improved through several cyclization methods including head-to-tail cyclization, side-chain cyclization, and peptide stapling (142, 143).

## 7 Computational pipeline for the selection, optimization, and translation of anticancer peptides from antimicrobial peptides

Early exploration experiments require enormous budgets because the identification and improvement of novel ACPs are labor-intensive, expensive, and time-consuming. In this context, bioinformatics tools present compelling methodologies for ACP discovery (144), that could be applied to enhance CAMPs, which are naturally anticancer, by making them more specific and potent with less toxicity to normal cells (145).

**FIGURE 4:**
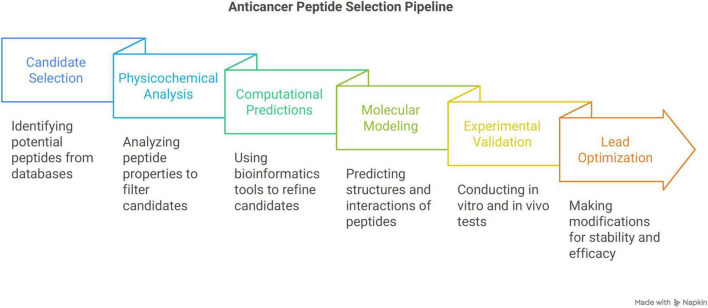
Anticancer peptides selection pipeline. The selection of ACPs from AMPs follows a structured method that begins with identifying potential candidates, then moves on to computational refinement and experimental validation, before advancing to clinical use.

The selection pipeline for ACPs from AMPs is organized systematically, encompassing phases of candidate selection, computational optimization, experimental validation, and clinical translation (Figure 4).

### 7.1 Selection of candidate peptides

The candidate selection process requires the evaluation of AMPs with anticancer activity retrieved from specialized databases such as APD3, CAMP, CAMP_*R4*_, DRAMP, DBAASP, and others. The evaluation includes assessment of key physicochemical properties including charge, hydrophilicity, hydrophobicity, amphipathicity, isoelectric point (pI) and propensity to *in vitro* aggregate (146–149). While removing toxic or hemolytic peptides using bioinformatic tools such as ToxinPred (150) and HemoPI (Table 2) (151–153), respectively.

**TABLE 2:** Computational tools for predicting ACPs.

Tool	Description	References
**ToxinPred**	Predicts peptide toxicity, including hemolytic potential, ensuring safety in peptide-based therapeutics.	(150)
**HemoPI**	Predicts the hemolytic potential of peptides, which is crucial for peptide-based therapeutic safety.	(151)
**HemoPred**	Web-based tool for assessing hemolytic properties of peptides to optimize safety in drug design.	(152)
**HAPPENN**	Predicts hemolytic activity of peptides using an ensemble neural network model.	(153)
**AntiCP**	Identifies ACPs based on amino acid composition and binary profile characteristics.	(154)
**AntiCP 2.0**	Enhanced version of AntiCP with improved predictive accuracy.	(154)
**ACPred**	Web-based tool designed to predict and characterize ACP activity in peptides.	(45)
**xDeep-AcPEP**	Uses deep learning to predict ACP activity and identify key functional residues.	(155)
**CancerGram**	Differentiates ACPs from AMPs and non-ACP/AMP peptides using a three-class model.	(156)
**TriNet**	Classifies ACPs with high accuracy using a deep neural network approach.	(157, 158)
**PEP-FOLD3**	Predicts 3D structures of peptides using a *de novo* approach based on fragment assembly simulations.	(159)
**AlphaFold 3**	AI-powered tool for protein and peptide structure prediction, integrating advanced deep learning techniques to model complex molecular interactions with high accuracy.	(160)
**GROMACS**	Molecular dynamics simulation package used to study peptide conformations, interactions, and stability.	(161)
**AMBER**	Suite of molecular simulation programs for modeling biomolecules, including peptides and proteins.	(162)
**CHARMM**	Molecular docking tool used to predict the interaction of peptides with target molecules.	(163)
**AutoDock**	Molecular docking tool used to predict the interaction of peptides with target molecules.	(164)
**HADDOCK**	Information-driven docking software for predicting protein-peptide and protein-protein interactions.	(165, 166)
**Rosetta**	Computational modeling suite for protein structure prediction, docking, and design of peptide therapeutics.	(167)
**PROSPER**	In silico tool for predicting protease-specific cleavage sites in peptides and proteins.	(169)
**PeptideCutter**	Predicts potential cleavage sites in a given peptide sequence for various proteases.	(170)

### 7.2 Computational optimization

The candidate peptides receive refinement through computational predictions and optimization which depends on bioinformatics tools such as AntiCP, ACPred and xDeep-AcPEP to evaluate anticancer activity (45, 154, 155). The classification tools such as CancerGram and TriNet server to distinguish antimicrobial from anticancer peptide (156–158).

Molecular modeling techniques predict peptide structures using tools such as PEP-FOLD3 and AlphaFold3 (159, 160), peptide-membrane interaction simulations are conducted with tools like GROMACS, AMBER, or CHARMM (161–163), and binding affinity assessment through docking studies using tools like AutoDock, HADDOCK or Rosetta (164–167).

Furthermore, Quantitative Structure–Activity Relationship (QSAR) models and AI-driven tools optimize sequences based on physicochemical correlations, machine learning algorithms (168), and stability predictions (PROSPER or PeptideCutter) (169, 170).

### 7.3 Experimental validation

Experimental validation involves conducting *in vitro* cytotoxicity assays, such as MTT colorimetric assay, assessing selectivity through hemolysis assays on erythrocytes, and evaluating toxicity on normal cells (171, 172). Additionally, membrane disruption assays, including flow cytometry, confocal microscopy, or calcein leakage assays, are examined (173).

Subsequently, *in vivo* validation is performed using tumor models, where applicable, encompassing efficacy evaluation through xenograft mouse models and toxicity assessment via hematological and histopathological analyses (174, 175).

### 7.4 Lead optimization and clinical translation

The phases of lead optimization and clinical translation necessitate modifications to improve stability, while formulation strategies are devised to ensure effective delivery. Preclinical evaluations concentrate on pharmacokinetics, immunogenicity, and scalability to facilitate the advancement of therapeutic development (176, 177).

## 8 Conclusion

Cationic antimicrobial peptides (CAMPs) are considered as potential anticancer agents because they can bind to the negative charged membrane of tumor cells without affecting normal cells. The anticancer activity of CAMPs is dependent on several physicochemical characteristics such as amino acid composition, net charge, hydrophobicity, amphipathicity and secondary structure. Examples such as KLA, bovine lactoferricin derivatives and LTX-315 have been shown to have potent anticancer activity, thus highlighting the potential of CAMPs as anticancer agents.

In order to improve the stability and bioavailability of CAMPs, several chemical modifications like terminal protection, backbone modification, glycosylation, PEGylation and cyclization have been used to enhance the resistance to proteolytic degradation. Moreover, to design next generation anticancer peptides, machine learning driven computational tools have been developed to improve the prediction. However, due to the existing problems such as high production costs, short half-life and possible off-target toxicity, CAMPs still stand as a valuable scaffold for the development of peptide-based anticancer therapeutics. Further structural optimization, chemical modifications and computational advancements will be crucial to fully exploit the potential of CAMPs for clinical uses.
